# VV‐ECMO for TRALI/TACO During Treatment of Hemorrhagic Gastric Ulcer: A Case Report

**DOI:** 10.1002/ccr3.71156

**Published:** 2025-10-07

**Authors:** Mayu Shindo, Hiroshi Matsuura, Hiroshi Hino, Masafumi Kishimoto

**Affiliations:** ^1^ Osaka Prefectural Nakakawachi Emergency and Critical Care Center Osaka Japan

**Keywords:** acute respiratory failure, endogenous hemorrhage, transfusion‐associated circulatory overload, transfusion‐related acute lung injury, veno‐venous extracorporeal membrane oxygenation

## Abstract

A 77‐year‐old man with no medical history was admitted with hematemesis. Vital signs indicated shock, and massive amounts of allogeneic blood were transfused. His respiratory condition deteriorated, and veno‐venous extracorporeal membrane oxygenation (VV‐ECMO) was initiated. He was weaned from VV‐ECMO and transferred to another hospital on Day 59.

## 1 Introduction

Diagnostic criteria for transfusion‐related acute lung injury (TRALI) were published at the Consensus Conference in 2004 [1] and were redefined in 2019 [2]. Diagnosis of the present case was based on these new criteria. The definition of transfusion‐associated circulatory overload (TACO) was decided on in 2018 [3]. TRALI and TACO are defined as hypoxemia that develops rapidly during or within 6 h after blood transfusion and that is associated with imaging evidence of bilateral lung infiltrates (Table 1) [2, 3]. The mortality rate of TRALI is estimated to be 5%–25%, especially in patients in the intensive care unit (ICU), and has been reported to be as high as 47% [4]. We report a case of TRALI/TACO after massive blood transfusion for shock associated with a bleeding gastric ulcer for which the patient was successfully treated with veno‐venous extracorporeal membrane oxygenation (VV‐ECMO).

**TABLE 1:** Diagnostic criteria for TRALI and TACO.

TRALI	TRALI Type I	Patients who have no risk factors for ARDS and meet the following criteria: i. Acute onset ii. Hypoxemia (P/F ≤ 300 or SpO_2_ < 90% on room air) iii. Cleare evidence of bilateral pulmonary edema on imaging (e.g., chest radiograph, chest CT, or ultrasound) iv. No evidence of left atrial hypertension (LAH) or, if LAH is present, it is judged to not be the main contributor to hypoxemia Onset during or within 6 h of transfusion No temporal relationship to an alternative risk factor for ARDS
	TRALI Type II	Patients who have risk factors for ARDS (but who have not been diagnosed with ARDS) or who have existing mild ARDS (P/F of 200–300), but whose respiratory status deteriorates and is judged to be due to transfusion based on: Findings as described in categories a and b of TRALI Type I, and Stable respiratory status in the 12 h before transfusion
TACO		Patients who have acute or worsening respiratory compromise and/or evidence of pulmonary edema (A and/or B below) during or up to 12 h after transfusion and the presence of a total of 3 or more of the criteria below: Acute or worsening respiratory compromise Evidence of acute or worsening pulmonary edema based on clinical physical examination, and/or radiographic chest imaging and/or other non‐invasive assessment of cardiac function (e.g., echocardiogram) Evidence for cardiovascular system changes not explained by the patient's underlying medical condition, including development of tachycardia, hypertension, widened pulse pressure, jugular venous distension, enlarged cardiac silhouette and/or peripheral edema Evidence of fluid overload including any of the following: a positive fluid valance; response diuretic therapy for example, from diuretic therapy or dialysis combined with clinical improvement; and change in the patient's weight in the peri‐transfusion period Supportive result of a relevant biomarker for example, an increase of B type natriuretic peptide level (e.g., BNP or NT‐pro BNP) above the age group‐specific reference range and greater than 1.5 times the pretransfusion value. A normal post‐transfusion NP level is not consistent with a diagnosis of TACO; serial testing of NP levels in the peri‐transfusion period may be helpful in identifying TACO
TRALI/TACO		Patients in whom TRALI cannot be distinguished from TACO or in whom both conditions occur simultaneously: Clinical findings compatible with TRALI and with TACO and/or lack of data to establish whether or not significant LAH is present

## 2 Case History/Examination

**FIGURE 1:**
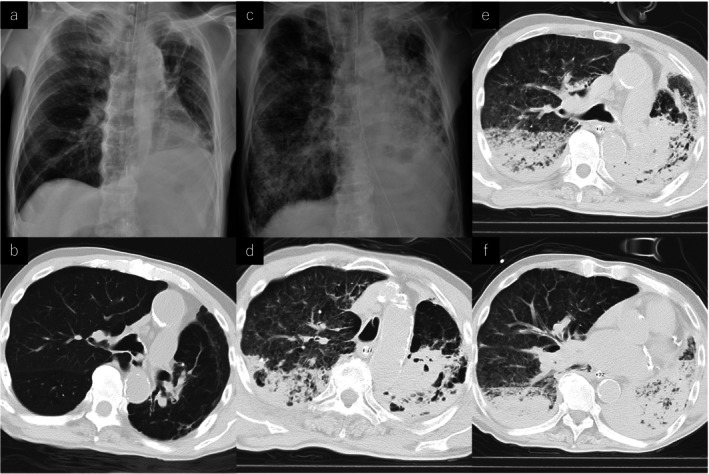
Chest X‐ray and chest computed tomography (CT) scans. (a) Chest X‐ray 30 min after the start of transfusion. (b) Chest CT scan on arrival (before transfusion). (c) Chest X‐ray 9 h after the start of transfusion. (d, f) Chest CT scans obtained at various lung levels 6 h after the start of transfusion.

**FIGURE 2:**
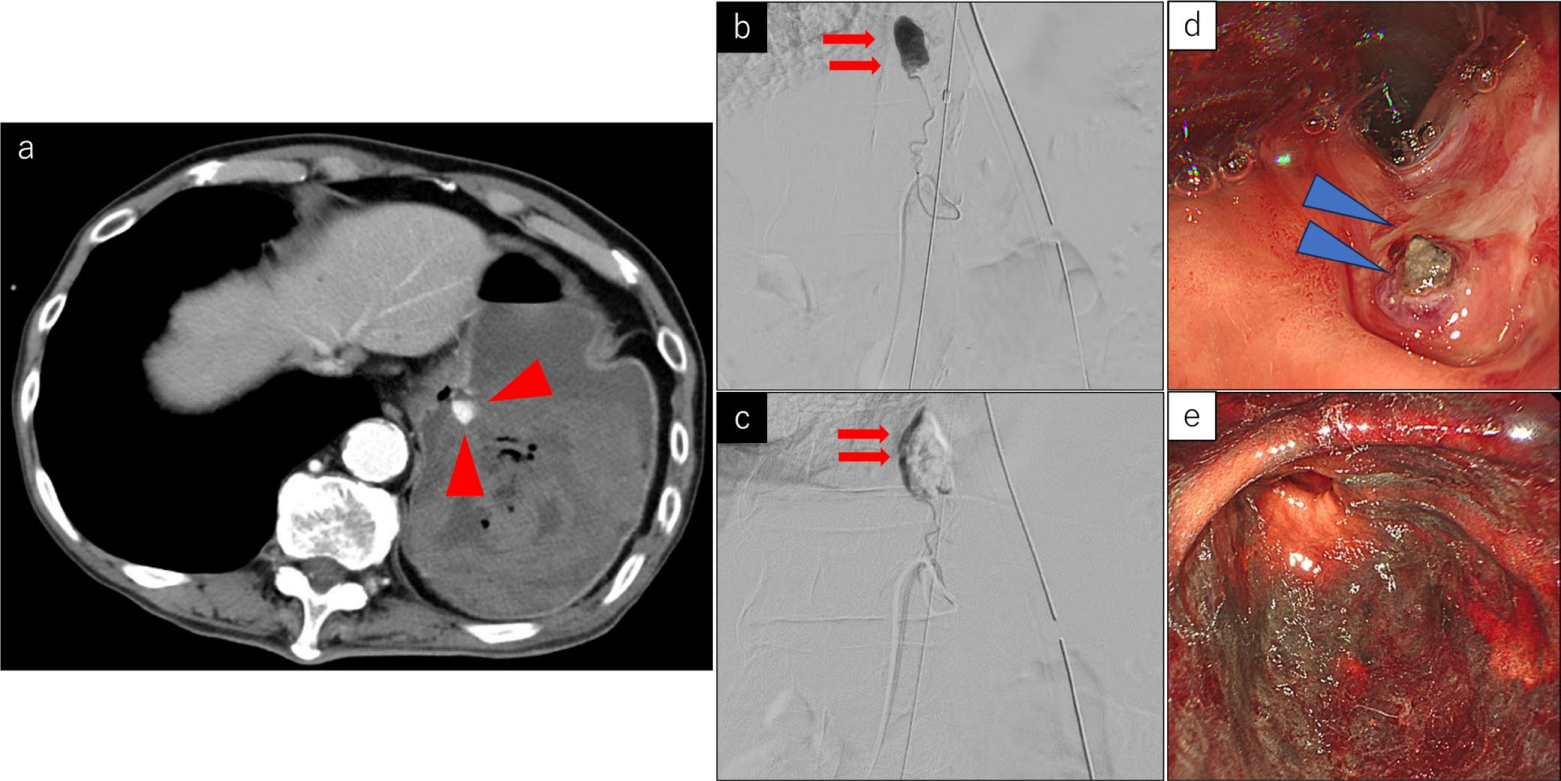
Contrast‐enhanced computed tomography (CT) scan on arrival, transcatheter arterial embolization (TAE), and upper endoscopy. (a) CT scan shows massive bleeding into the stomach. Red arrowheads show a large amount of extravascular leakage into the stomach. (b) Before TAE to the left gastric artery branch (red arrows). (c) After TAE to the left gastric artery branch (red arrows). d: Gastric ulcer with exposed blood vessels (blue arrowheads). e: The stomach showing ischemic changes.

**FIGURE 3:**
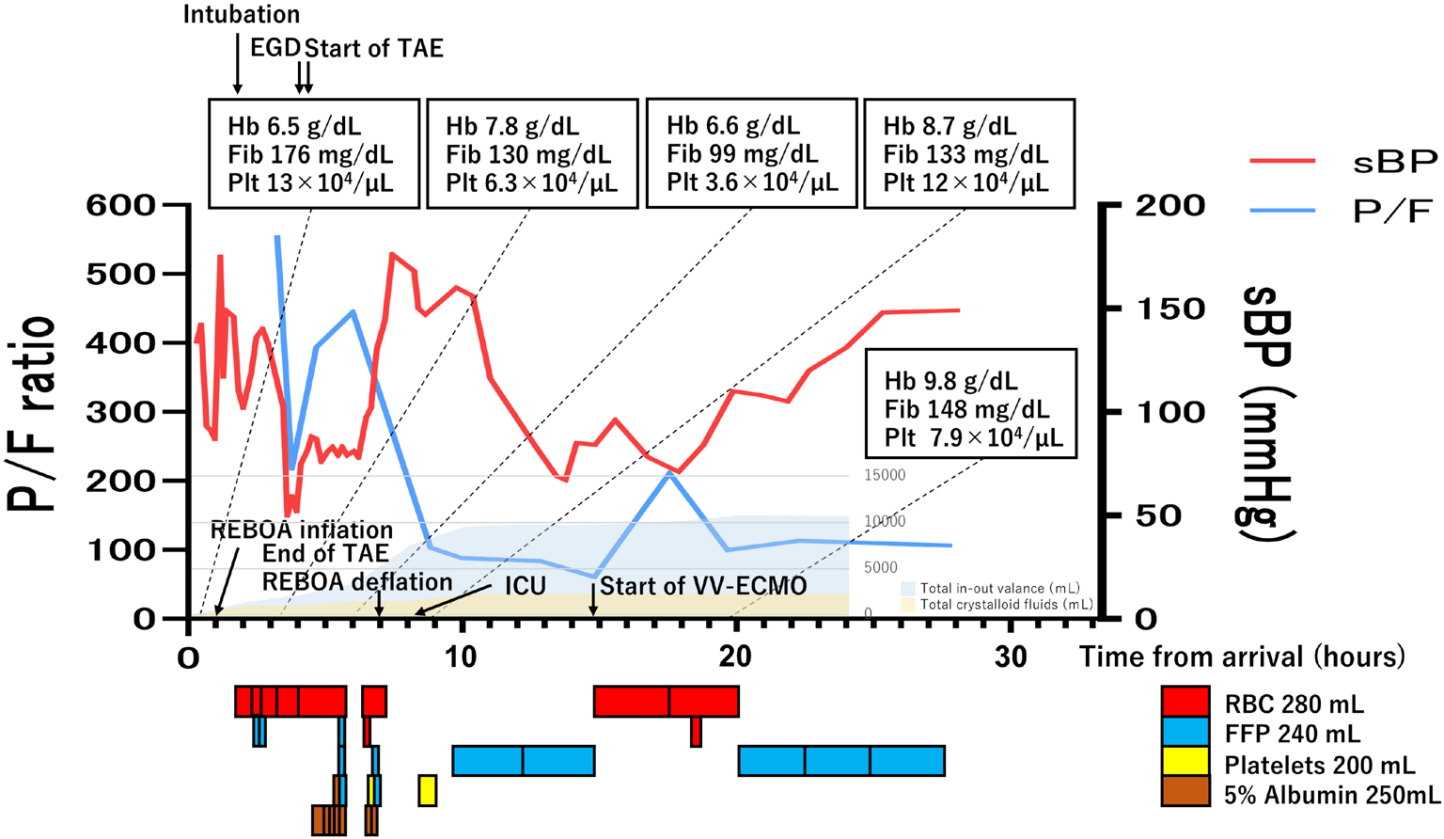
Clinical course of the patient. EDG, Esophagogastroduodenoscopy; FFP, Fresh frozen plasma; Fib, Fibrinogen; Hb, Hemoglobin; ICU, Intensive care unit; Plt, Platelets; RBC, Packed red blood cells; REBOA, Resuscitative endovascular balloon occlusion of the aorta; TAE, Transcatheter arterial embolization; VV‐ECMO, Veno‐venous extracorporeal membrane oxygenation.

A 77‐year‐old man with no medical history (height, 170 cm; weight, 61 kg) was transported to our emergency center for hematemesis and severe general malaise. His vital signs suggested shock, and gastrointestinal bleeding was suspected due to dark red stools on admission. His Glasgow Coma Scale was 12 (E3V4M5), heart rate was 100/min, blood pressure was 125/45 mmHg, and SpO_2_ was 100% on O_2_ at 10 L/min. His blood pressure decreased without extracellular fluid administration. Laboratory findings on admission are shown in Table 2. No data indicated liver or renal dysfunction. Chest radiography showed no bilateral lung field opacities or cardiac enlargement (Figure 1a). An enhanced computed tomography (CT) scan showed a large amount of extravascular leakage into the stomach, but there were no lung abnormalities (Figures 1b and 2a). The patient was administered histamine‐2 receptor antagonists. We attempted to control the bleeding with resuscitative endovascular balloon occlusion of the aorta (REBOA) in zone 1 before performing gastrointestinal endoscopy and transcatheter arterial embolization (TAE). As his vital signs were worsening, partial inflation of REBOA was performed at 4–16 mL while monitoring right radial artery pressure. After endotracheal intubation, we performed upper gastrointestinal endoscopy but could not identify the source of bleeding due to a large hematoma in the stomach. TAE was performed in the left gastric artery branch to stop the bleeding (Figure 2b,c). The patient remained hemodynamically unstable until hemostasis was achieved and required blood transfusions and a large amount of 5% albumin (Figure 3). Transfusion of concentrated red cells (1960 mL), fresh frozen plasma (1680 mL), platelet concentrate (200 mL), and 5% albumin were administered before ICU admission, and concentrated red cells (840 mL), fresh frozen plasma (720 mL), and platelet concentrate (200 mL) were administered after ICU admission. Only RhD‐negative allogeneic transfusion products were used.

**TABLE 2:** Laboratory findings on admission.

**Venous blood gas analysis (FiO_2_: 0.21)**		
pH	7.203	
Hemoglobin (Hb)	6.7	g/dL
Base excess (BE)	−10.4	mEq/L
HCO_3_^−^	16.3	mmol/L
Lactate	10.9	mmol/L
**Blood tests**		
RBC	1.87	million/μL
Hb	6.5	g/dL
Hematocrit	21.3	%
Platelets	1.34 × 10^5^	/μL
WBC	11,740	/μL
PT%	105	%
PT‐INR	0.98	
APTT	25.2	sec
Fibrinogen	176	mg/dL
FDP	2.8	μg/mL
D‐dimer	2.28	μg/mL
Blood type	A	
RhD	Negative	
Irregular antibodies	Negative	

## 3 Differential Diagnosis, Investigations, and Treatment

The patient's hemodynamic status stabilized after TAE, but his respiratory status began to deteriorate. At the end of TAE, the REBOA was deflated and removed. It was worsening bilateral infiltrates in the subsequent chest radiography (Figure 1c), and a CT scan showed infiltrative shadows and pleural effusions in both lungs (Figure 1d–f). After the CT scan, the patient was admitted to the ICU due to the deterioration in his condition.

Approximately 7 h after the first transfusion and 1.5 h after the end of the last transfusion, his PaO_2_/FiO_2_ (P/F) ratio was 100, PaCO_2_ was 50 mmHg, and respiratory settings on mechanical ventilation were set to FiO_2_ 100%, PEEP 12 mbar, and inspiratory pressure 26 mbar. Echocardiography revealed a left atrial diameter of 33 mm, left ventricular end‐diastolic diameter of 48 mm, left ventricular end‐systolic diameter of 37 mm, left ventricular contractility of 41%, and wall thinning and abnormal wall motion from the anterior septal base to the apex. His lactate level decreased after hemostasis, even though the ventilator was set to FiO_2_ 100%, high‐pressure phase 28 mbar, low‐pressure phase 0 mbar, high‐pressure time 5 s, low‐pressure time 0.5 s on airway pressure release ventilation. His arterial blood gas test showed a P/F ratio of 60, PaCO_2_ 87 mmHg, pH 7.103, and HCO_3_^−^ 21.2 mmol/L. Steroids and antimicrobials were started for suspected acute respiratory distress syndrome and pneumonia, but the clinical course of the patient led us to diagnose TRALI/TACO. As shown in Figure 3, his blood pressure gradually decreased after admission to the ICU. Lung‐protective ventilation failed, leading to refractory hypoxemia; VV‐ECMO was initiated approximately 16 h after admission for resuscitation of the respiratory failure. A 24 Fr access cannula was inserted via the right femoral vein and a 20 Fr access cannula was inserted via the right internal jugular vein (femoro‐jugular configuration). FdO_2_ was 100%, sweep gas was 3 L/min, and flow was 3.85 L/min with 1700 rpm. The patient had gastrointestinal bleeding and was started on VV‐ECMO without anticoagulants. Total fluid balance on Day 1 was +10,662 mL, but his blood pressure decreased and he needed vasopressors; it was difficult to use diuretics on Day 1. As his vital signs stabilized, diuretics were started on Day 2. Upper gastrointestinal endoscopy on Day 4 revealed the source of bleeding to be a hemorrhagic gastric ulcer, and his stomach showed overall ischemic changes (Figure 2d,e). Hemostasis had been achieved; systemic anticoagulation with nafamostat mesylate was started on Day 5. His lung permeability on chest radiography improved over time. An ECMO weaning trial was performed on Day 8 but was not successful. On Days 9 and 10, hemorrhage occurred from the transverse pancreatic artery and the posterior gastric artery, respectively, and TAE was performed in both arteries. During embolization of the transverse pancreatic artery, blood flow to the pancreas was partially reduced, and pancreatitis occurred. An intra‐abdominal hematoma was formed on the dorsal gastric wall. Drains were placed under the left diaphragm and in the Douglas fossa. The intra‐abdominal hematoma formed on the dorsal gastric wall traversed the stomach and the main pancreatic duct and shrank over time with insertion and drainage of a gastric tube.

## 4 Results (Outcome and Follow‐Up)

On Day 11, the patient was successfully weaned from ECMO, and a tracheostomy was performed. After the withdrawal of ECMO, atelectasis easily formed that required bronchoscopic expectoration, but his respiratory status was maintained with a ventilator. Although the patient was able to communicate, weaning from the ventilator was difficult, and he was transferred to a convalescent hospital on Day 59.

## 5 Discussion

Our patient presented with a GI bleed, received multiple transfusions, and subsequently developed TRALI/TACO. Despite the patient's good respiratory status on admission, his P/F ratio was 100 at 1.5 h after the end of blood transfusion, and chest radiography and CT showed marked infiltrative shadows with findings of pulmonary edema. The patient had no underlying cardiovascular disease or left atrial enlargement after the onset of respiratory failure, but he did have a high NT‐proBNP level of 2003 pg/mL. Based on his clinical course, we diagnosed the patient as having TRALI/TACO according to the TRALI and TACO evaluation criteria [2, 3]. The patient had a high Acute Physiology and Chronic Health Evaluation II score of 20 at the time of admission, was elderly, and had undergone TAE requiring frequent blood transfusions. None of the risk factors for TACO were present [5].

This patient required several additional TAE procedures after hemostasis for a bleeding gastric ulcer. He had no history of oral NSAID use to cause risk of ulcer and no evidence of *Helicobacter pylori* infection on endoscopy. The patient had a Dieulafoy ulcer. There was no evidence of vasodilatation or stenosis on imaging, but he had extensive atherosclerotic lesions with intimal calcification. The patient had vascular fragility against a background of atherosclerotic lesions, which was thought to be a combination of various factors leading to the recurrent arterial bleeding. The patient required large blood transfusions during the bleeding episodes, and it was difficult to manage fluids for weaning from ECMO. Moreover, the patient was 77 years old and had mild emphysema on the admission CT scan that led to the progression of disuse syndrome, hypoventilation, and pneumonia due to respiratory muscle weakness, and difficulty in weaning from the ventilator.

Only four patients with severe respiratory failure due to TRALI have been reported to have a good outcome with VV‐ECMO. Their characteristics are summarized in Table 3. The time from transfusion to onset ranged from 40 to 120 min, and from VV‐ECMO to withdrawal ranged from 16 h to 6 days. Various transfusion products were administered and background diseases were present [6, 7, 8, 9]. The mortality rate of TRALI in the ICU is as high as 47%. However, there are cases in which VV‐ECMO has saved lives. To our knowledge, there are no reports of the use of VV‐ECMO for severe respiratory failure due to TACO or TRALI/TACO. This case suggests that VV‐ECMO may also save lives in TRALI/TACO complicated by hemorrhagic disease. In cases of TRALI/TACO with severe respiratory failure, early recognition and treatment are important to reduce patient mortality. The factors associated with TRALI/TACO severity and prognosis are unclear, and further case studies are needed.

**TABLE 3:** Characteristics of patients with severe respiratory failure due to TRALI who were successfully treated with VV‐ECMO.

Year	Author	Age	Sex	Diagnosis	Procedures	History	Transfusion	Time to onset after transfusion	Duration of VV‐ECMO	Duration of mechanical ventilation
2009	Kuroda H	58	M	TRALI	Right‐lobe hepatic resection for hepatocellular carcinoma	Child‐Pugh class A	RBC 280 mL, FFP 900 mL	120 min	2 days	6 days
2014	Stan R	16	F	TRALI	Primary reconstructive surgery for craniofacial injury	Unstable C5‐arch fracture and dislocated right humerus fracture	RBC 2240 mL, FFP 1440 mL, PC 20 mL	45 min	16 h	2 days
2015	Honda A	70	F	TRALI	Laparoscopic nephrectomy	No data	Several units of RBC and FFP	Shortly thereafter	No data	No data
2023	Ishida Y	79	M	TRALI Type I	Total cystectomy and ileal conduit diversion for bladder cancer	Acute myeloid leukemia, thoracic aortic aneurysm, myocardial infarction	RBC 560 mL, PC 400 mL	40 min	6 days	7 days
2024	Ours	77	M	TRALI/TACO	TAE for hemorrhagic gastric ulcer	No medical history, Rh negative	RBC 1960 mL, FFP 1680 mL, PC 200 mL	90 min	11 days	Not removable

Abbreviations: FFP, fresh frozen plasma; PC, platelet concentrate; RBC, red blood cells; TACO, transfusion‐associated circulatory overload; TAE, transcatheter arterial embolization; TRALI, transfusion‐related acute lung injury; VV‐ECMO, veno‐venous extracorporeal membrane oxygenation.

## 6 Conclusion

We experienced a case of TRALI/TACO after blood transfusion for bleeding gastric ulcer that was successfully treated with VV‐ECMO. Further case accumulation is needed to elucidate the pathophysiology of TRALI/TACO and establish treatment methods.

## Author Contributions

**Mayu Shindo:** conceptualization, investigation, writing – original draft, writing – review and editing. **Hiroshi Matsuura:** conceptualization, supervision, writing – original draft, writing – review and editing. **Hiroshi Hino:** conceptualization, supervision. **Masafumi Kishimoto:** conceptualization, supervision.

## Ethics Statement

The authors have nothing to report.

## Consent

We obtained written informed consent from the patient and his family for publication of this case report.

## Conflicts of Interest

The authors declare no conflicts of interest.

## Data Availability

The patient's data is available from the corresponding author on reasonable request.
